# Dialysis adequacy revisited: Kt/V's blind spot for phosphorus and iodine

**DOI:** 10.1515/jtim-2025-0050

**Published:** 2025-10-25

**Authors:** Changhao Zhu, Cheng Xue, Naiying Lan, Fanzhou Zeng, Hao Wang, Bo Yang

**Affiliations:** Department of Nephrology, Naval Medical Center of PLA, Naval Medical University, Shanghai, China; Division of Nephrology, Shanghai Changzheng Hospital, Second Affiliated Hospital of Naval Medical University, Shanghai, China

The Kt over V (urea) (Kt/V) index is the current standard for assessing the adequacy of maintenance hemodialysis (MHD), primarily reflecting the clearance of small molecules like urea. However, its focus on a single solute overlooks substances with different and more complex biokinetics. This perspective article highlights this deficiency using phosphorus and iodine as key examples. Both substances are characterized by a three-compartment distribution model, with large reservoirs in bone and the thyroid gland, respectively. Consequently, standard dialysis sessions, even when achieving target Kt/ V values, may fail to adequately clear total body phosphorus and iodine, leading to significant post-dialysis rebound and contributing to morbidities such as hyperphosphatemia and potential thyroid dysfunction. We argue that relying solely on Kt/ V creates a blind spot in dialysis prescription. We call for a more nuanced understanding of dialysis adequacy, urging physicians to consider the specific pharmacokinetic models of key uremic toxins. Further research into multi-compartment modeling is necessary to develop complementary metrics to better guide dialysis therapy and improve patient outcomes.

Adequate dialysis forms the cornerstone of ensuring a high quality of life and favorable long-term prognosis for patients undergoing maintenance hemodialysis (MHD).^[1]^ Currently, the assessment of hemodialysis adequacy primarily relies on the Kt/ V index. While Kt/V is acknowledged as an effective measure for evaluating the clearance of small-molecule metabolic products, such as urea, during a single dialysis session, it exhibits several inherent limitations. Indeed, while some defend its continued utility, these limitations warrant critical examination. Kt/ V relies on a single solute, urea, as a representative marker. This may not accurately reflect the clearance of other potentially toxic molecules with differing kinetic profiles. The focus on urea might overlook the accumulation of other uremic toxins, which also contribute to adverse outcomes in MHD patients. In patients with smaller body sizes or those who are malnourished, Kt/ V may overestimate the adequacy of dialysis. The volume of distribution (V) in the Kt/V formula is influenced by body size, potentially leading to an artificially elevated Kt/ V value in smaller individuals, even if the actual clearance is suboptimal.^[2]^ This highlights the need for caution when interpreting Kt/V in patients with atypical body compositions. The Kt/ V formula was initially developed and applied during an era when dialysis membranes had smaller surface areas and pore sizes. Modern high-flux dialyzers offer enhanced clearance capabilities, rendering the original assumptions of the Kt/ V formula potentially outdated.^[3]^ The improved efficiency of contemporary dialysis membranes necessitates a reevaluation of the appropriateness of Kt/ V as a sole marker of dialysis adequacy.

Despite its known flaws, Kt/V persists as the primary measure of dialysis adequacy, highlighting the need for alternative metrics. This article further explores its limitations, focusing on its failure to accurately reflect the clearance of substances with specific biokinetics, such as phosphorus and iodine.

Chronic kidney disease (CKD) patients, both pre-dialysis and on dialysis, exhibit a high incidence of hyperphosphatemia. This condition is inextricably linked to the diminished renal function, leading to phosphate retention. While phosphorus, present in the blood as phosphate, can be cleared *via* hemodialysis, in practice, patients undergoing conventional hemodialysis often find it challenging to achieve adequate serum phosphorus control through increased dialysis dosage alone. This difficulty persists even when conventional measures of dialysis dose, such as Kt/V, are met or exceeded. Highlighting this gap, Levin *et al*. reported that the goal of normalizing phosphorus levels is “rarely achieved” even with “regular and adequate dialysis treatment”, with data showing that over a third of patients remain hyperphosphatemic.^[4,5]^ This demonstrates that dialysis adequacy, as measured by urea clearance, does not guarantee control of phosphorus. The management of hyperphosphatemia typically necessitates a combination of dietary restrictions and the administration of phosphate binders. The underlying reason for this phenomenon lies in the distinct biokinetics model of phosphorus, which differs from that of urea and creatinine. Unlike the one- or two-compartment models of typical small molecule metabolites, phosphorus exists within a larger, “third compartment”: bone and teeth. Approximately 1% of human body weight is comprised of phosphorus, with 85% distributed in bone and teeth, 14% in soft tissues, and only 1% in extracellular fluid, including plasma.^[6]^ During a standard 4-hour hemodialysis treatment, even with efficient solute exchange between dialysate and plasma, only a small fraction of the total body phosphorus is accessible for removal. Each standard dialysis session clears approximately 900 mg of phosphorus. However, the phosphorus that has been cleared is rapidly replenished from the extensive skeletal reservoir during the interdialytic period. The presence of this third compartment explains the difficulty in effectively treating hyperphosphatemia through dialysis optimization alone.

**Figure 1:**
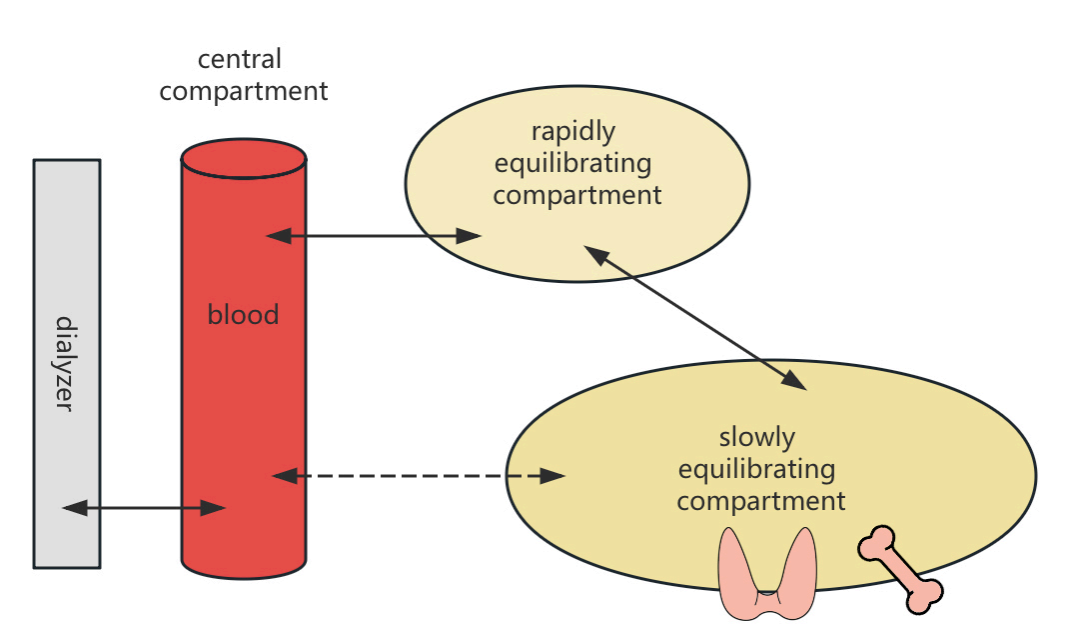
Graphic illustration of the three-compartment model.

Iodine is another clinically important substance, as healthy kidneys excrete approximately 90% of it through urine.^[7]^ Patients with impaired renal function experience reduced iodine excretion. The typical dialysis dose for MHD patients does not meet the physiological excretory demands for iodine. Direct evidence indicates that the effective half-life of ^131^I in MHD patients is 44 h, which is 2.39 times longer than that in individuals with normal renal function.^[8]^ Although iodine’s ionic form and low molecular weight suggest it should be easily cleared by dialysis, its removal is complicated by extensive storage in a “third compartment”. The thyroid gland alone holds 70%–80% of the body’s iodine, with significant amounts also found in the mammary glands, eyes, and gastrointestinal mucosa. The iodine present in the blood constitutes only a small fraction of the total body iodine. During a standard 4-hour hemodialysis treatment, even with efficient solute exchange between dialysate and plasma, the re-equilibration between the deep and shallow peripheral compartments, as well as the central compartment, leads to a significant rebound in iodine levels (Figure 1). As illustrated in Figure 1, the dialyzer removes solutes directly from the central compartment (blood). Following dialysis, the concentration gradient drives solutes from the rapidly and slowly equilibrating peripheral compartments (*e.g*., soft tissues, thyroid gland) back into the blood. This process of re-equilibration is responsible for the clinically observed post-dialysis rebound in solute levels, a dynamic not captured by the single-compartment-based Kt/ V formula. The inability of dialysis to adequately clear iodine has clinical implications, although this issue has not yet garnered sufficient research attention. Evidence suggests a high prevalence of thyroid diseases, primarily hypothyroidism, in MHD patients.^[9]^ While the cited evidence establishes an association rather than direct causality, the potential role of iodine retention warrants consideration, though a direct causal link has not yet been established. It is hypothesized that chronic iodine excess may contribute to the observed thyroid dysfunction in this population. Pathophysiologically, sustained high levels of intrathyroidal iodine can induce the Wolff-Chaikoff effect, an autoregulatory phenomenon that transiently inhibits thyroid hormone synthesis and release. In uremic patients with impaired iodine excretion, this effect may become prolonged, potentially leading to overt hypothyroidism.^[10]^ This hypothesis of iodine-induced thyroid dysfunction warrants further investigation, as the inability of dialysis to adequately clear total body iodine has not yet garnered sufficient research attention. Currently, dietary recommendations to restrict iodine intake and other strategies to reduce iodine exposure in MHD patients are lacking. However, with the accumulation of further evidence, this issue may gain prominence in the future.

Phosphorus and iodine exemplify compounds with a similar three-compartment distribution kinetic model (Figure 1), characterized by large apparent volumes of distribution. The dialysis clearance of these substances cannot be accurately described by the existing Kt/V formula. Moreover, when considering whether to implement extracorporeal enhancement for certain intoxications, the pharmacokinetic model of the drug must be taken into account. Toxins with smaller molecular weights, small apparent volumes of distribution, and low protein binding rates are more likely to exhibit better clearance rates following dialysis treatment.

In light of the aforementioned limitations of Kt/ V in describing dialysis adequacy, we recommend further investigation into distributed modeling of diffusive solute with typical three-compartment distribution kinetic structures in hemodialysis. The goal of such research should be to develop and validate supplementary clinical indicators that account for multi-compartment kinetics. For example, metrics such as a toxin-specific “rebound index” or an extended clearance formula could be developed. Simultaneously, hemodialysis prescription physicians should possess a correct understanding of Kt/ V, incorporating the distribution model of the substance to be cleared into their considerations when prescribing dialysis, and forming more reasonable expectations regarding the effectiveness of dialysis treatment. The clinical integration of these complex models could be validated against established serum markers by tracking solute rebound over extended interdialytic periods.
